# The impact of digital tools and the expected digital transformation in radiotherapy on Dutch radiation therapists (RTTs)

**DOI:** 10.1016/j.tipsro.2025.100319

**Published:** 2025-06-04

**Authors:** Thijs Ackermans, Paul Cremers, Janne Dingemans, Carol Ou, Marcel Verheij, Maria Jacobs

**Affiliations:** a Department of Radiation Oncology (Maastro), GROW Research Institute for Oncology and Reproduction, Maastricht University Medical Centre+, Maastricht, the Netherlands; b Tilburg School of Economics and Management, Tilburg University, Tilburg, the Netherlands; c Department of Radiation Oncology, Radboud University Medical Center, Nijmegen, the Netherlands

**Keywords:** Radiotherapy Technologists, Digital tools, Technostress, Digital Transformation, Job Performance Indicators, Job Autonomy

## Abstract

**Background and Purpose:**

Technological advancements have the potential to mitigate RTT shortages, burnout levels, and turnover rates. However, it is unclear if RTTs currently experience technostress (stress or anxiety from being overwhelmed by technology) and how extensive digital tool usage impacts key job performance indicators (e.g., job engagement, satisfaction, and turnover intentions). This study investigates the impact of digital tools used and expected digital transformation on RTTs’ technostress and key job performance indicators.

**Materials and Methods:**

A survey was distributed to RTTs across all Dutch radiotherapy centers, resulting in 265 respondents. The survey included validated questionnaires on digital tool use, technostress, job autonomy, innovative work behavior and job performance indicators. Relationships were assessed using linear regression models and paired samples T-tests (p < 0.05).

**Results:**

The number of digital tools used had no significant effect (p < 0.05) on key job performance indicators and was not related to technostress. Technostress negatively impacted key job performance indicators, but explained less than 7 % of the variance. Other factors, such as job autonomy, had a greater influence. When presented with a description of the future perspective of their job role after the expected digital transformation, RTTs reported small declines in key job performance indicators.

**Conclusion:**

Current RTT performance is not affected by the number of digital tools used, as RTTs are used to working with digital tools. RTTs showed no technostress. After the predicted digital transformation RTTs remained satisfied and engaged. Ensuring RTTs feel skilled and empowered with decision-making authority is crucial before introducing new digital tools.

## Highlights

• The performance of the RTT is not affected by the number of digital tools used.
• The Dutch RTTs show no indications of technostress.
• The predicted digital transformation did not impact key job performance indicators.

## Introduction

Radiotherapy is a highly effective cancer treatment modality that has benefited from technological advancements to identify and target tumors with high accuracy and precision [1]. However, like other care domains, the field of radiotherapy is impacted by staffing shortages, posing significant challenges on radiation therapists (RTTs) to maintain high-quality care delivery [2,3]. Furthermore, the physical and psychological strain associated with working in oncology only exacerbates the staff shortage problem, resulting in high levels of burnout and relatively high staff turnover, and hence a limited average employee retention rate [[4], [5], [6], [7]]. It is important to anticipate on these challenges in radiotherapy when considering implementing emerging innovations.

Technological advancements, as part of the ongoing digital transformation (which can be defined as “a process that aims to improve an entity by triggering significant changes to its properties through combinations of information, computing, communication, and connectivity technologies” [8]), have the potential to both enhance care quality and counteract the high levels of burnout and staff turnover [3,9]. Presently, RTTs use digital tools for a large portion of their work (such as treatment planning (e.g. automatic planning, automatic contouring or plan optimalization) [10,11], patient positioning [12], communication and reporting [11,12]) and each tool might have a different impact. Consequently, technological advancements that enhance RTTs’ efficiency have the potential to positively impact various aspects of their daily routine [[11], [12], [13], [14]].

Although the extensive use of digital tools may enhance the efficiency of RTTs, it may induce technostress (i.e. anxiety, tension, or distress caused when a person is overwhelmed by technology) among RTTs [15]. Despite of its importance, it is currently under explored if RTTs experience technostress caused by the increasing use of digital tools and, additionally, whether the experienced technostress has an impact on the key job performance indicators (e.g. Job Engagement, Job Satisfaction and Turnover Intentions) [[16], [17], [18]].

Another potential drawback is the relatively rapid pace of the digital transformation in radiotherapy in the Netherlands, which could lead to resistance of the RTTs [19]. Moreover, the digital transformation will most likely result in the automation of tasks traditionally performed by RTTs [20]. For instance, while RTTs currently spend up to 50 % of their time on treatment planning, AI systems could simplify or perhaps even make this part of their job redundant [10]. This evolution of responsibilities could also lead to dissatisfaction and resistance among RTTs, potentially driving the high turnover rates and low retention levels. As a result, decision-makers in radiotherapy are often hesitant to implement another digital tool due to the potential risk of employee resistance. However, it should be noted that some individuals embrace the digital transformation instead of showing signs of technostress or resistance these individuals (i.e. individuals displaying innovative work behavior [21] or perceiving high levels of Job Autonomy [22]) show an improvement in the key job performance indicators [15].

**Fig. 1:**
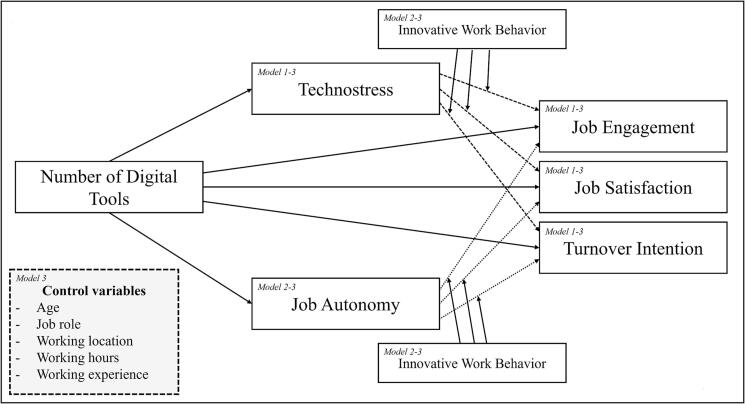

**Fig. 2:**
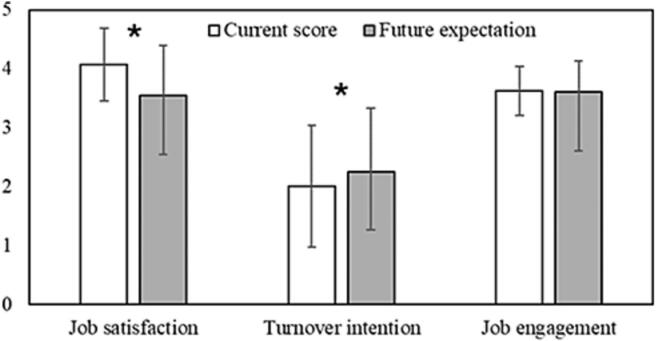

The overall aim of the present study is therefore to investigate 1) the impact of the currently used digital tools on specific RTT’s key job performance indicators (Job Engagement, Job Satisfaction and Turnover Intentions (Fig. 1)) and 2) the impact of the expected digital transformation in radiotherapy on the RTT’s key job performance indicators. In addition, we will investigate if the Technostress, Job Autonomy and innovative work behavior the RTT influences these relationships (Fig. 1). This approach will help us identify the factors driving potential employee resistance or Turnover Intentions amongst RTTs when implementing a new digital tool. Additionally, it will enable us to evaluate strategies for overcoming these challenges and ensuring smoother implementation of digital tools. Moreover, these insights will contribute to ensuring the future sustainability of radiation therapy care in the Netherlands.

## Materials and Methods

### Participants and survey distribution

The target population of the structured self-reporting survey consisted of all RTTs working in the Netherlands (approximately N = 800, working across 19 radiotherapy centers). The survey, based on validated questionnaires, was distributed between April and May 2024 using two approaches: 1) an invitation was published in the monthly newsletter and website of the Dutch Society for Medical Imaging and Radiotherapy (Nederlandse Vereniging Medische Beeldvorming & Radiotherapie [23]) and; 2) an email invitation was sent to the medical chairs of the different radiotherapy centers in the Netherlands. A link to the online survey was attached to both invitations, along with information for the participants that emphasized the voluntary nature of survey participation and assured them of confidentiality. A follow-up reminder was sent to the medical chairs approximately three weeks later.

### Survey outline

The survey consisted of a total of 94 mandatory questions categorized into the following sections (Supplementary Table 1):

• **Participant characteristics.** The following personal characteristics were questioned in the first section of the survey: Age (18–25 years (y); 26-35y; 36-45y; 46-55y; or > 55y), Gender (male; female; or other), Job role (all-round RTT, Planning RTT, machine RTT or other), Working experience (0-5y; 6-10y; 11-15y; 16-20y; or > 20y), Working hours (0–8 h (h); 9–16 h; 17–24 h; 25–32 h; or 33-40y) and Working location (open question to be completed by RTT).
• **Innovative behavior in the workplace** was questioned using the validated nine-item innovative work behavior (IWB) questionnaire, with a five-point Likert scale (1 = strongly disagree; 2 = disagree; 3 = neutral; 4 = agree; 5 = strongly agree) (21). Example statement of the IWB questionnaire: ‘I invent new ideas for difficult problems’. The overall IWB score was calculated as the mean of all nine items.
• **Job Autonomy** was questioned using the validated 23-item work design questionnaire [24] that was scored on a five-point Likert scale (1 = strongly disagree; 2 = disagree; 3 = neutral; 4 = agree; 5 = strongly agree). Example statement of the questionnaire: ‘My current job allows me to make my own decisions about how to schedule my work.’
• **Digital tools.** The RTTs were asked whether or not they used digital tools to perform 10 different routine tasks: 1) Software to automate the preparation phase (e.g. scripting); 2) Software to automatically import CTs; 3) Software to optimize the treatment plan; 4) Software to automatically position the patient; 5) Automatic contouring software; 6) Software to automate the development of the treatment plan; 7) Software to automatically detect deviations; 8) Software for automatic reporting (e.g. data coupling); 9) Software to automatically make a 3D reconstruction; 10) The use of an electronic medical record. The intensity that the RTT used each task was scored on a five-point Likert scale (1 = never; 2 = rarely; 3 = sometimes; 4 = often; 5 = always).
• **Technostress.** To determine the presence of technostress the validated 14-item technostress questionnaire was included in the survey [15]. Each item was scored on a five-point Likert scale (1 = strongly disagree; 2 = disagree; 3 = neutral; 4 = agree; 5 = strongly agree). The overall technostress was calculated as the mean score of all items. In addition, the following variables were calculated: 1) Techno overload (mean of 5 items (e.g. ‘I am forced by digital tools to work much faster’); 2) Techno complexity (mean of 5 items (e.g. ‘I do not know enough about the new digital tools to handle my job satisfactorily’); 3) Techno insecurity (mean of 3 items (e.g. ‘Because of new digital tools, I feel constant threat to my job security’); 4) Techno uncertainty (1 item: ‘In our organisation, there are always new developments in the digital tools we use’).
• **Job performance indicators.** To get an indication of job performance of the RTT, the following aspects were questioned using different validated questionnaires:
  1. Job Satisfaction. Single item scored on a five-point Likert scale (1 = strongly disagree; 2 = disagree; 3 = neutral; 4 = agree; 5 = strongly agree) from Johnston & Lee [25]:’All things considered, I’m satisfied with my current job’.
  2. Job Engagement. Assessed using a nine-item questionnaire scored on a five-point Likert scale (1 = strongly disagree; 2 = disagree; 3 = neutral; 4 = agree; 5 = strongly agree) [15]. Example statement of the questionnaire: ‘I am proud of the work that I do’. The overall Job Engagement was calculated as the mean score of all items [15].
  3. Turnover Intention. Assessed using a single question adopted from Mitchell et al [26] and scored on a five-point Likert scale (1 = strongly disagree; 2 = disagree; 3 = neutral; 4 = agree; 5 = strongly agree). Statement was as follows: ‘I am considering quitting the position of RTT’.
• **Future perspective.** The RTT was presented with a future (approximately 10 years from now) view of their role (i.e. taking the expected technological advancements into account). In short, the future perspective highlighted that RTTs 1) would have less patient interaction; 2) that large portion of treatment planning would be automated; 3) the RTT’s role would focus more on quality control and monitoring; 4) fewer manual interactions would be required in treatment delivery potentially reducing the number of RTTs per device. The input for this future perspective on the RTT’s role was provided by the literature and experts working in the field (i.e. five RTT’s, head of medical physics, CEO of a radiotherapy center, care manager and manager research) [[27], [28], [29]]. In order to investigate how the RTTs anticipate the proposed technological advancements changed their level of Job Engagement, Job Satisfaction and Turnover Intention we used the same questions as outlined before.

### Statistical analysis

#### Current situation

For each RTT the count of digital tools was obtained by counting the number of tools (out of the 10 listed tools) that were used ‘often’ or ‘always’. Three linear regression models assessed the predictive value of the count of digital tools to predict 1) the key job performance indicators (Job Satisfaction, Job Engagement and Turnover Intention); 2) the overall technostress score; and 3) Job Autonomy. Next, the RTTs were grouped into two groups (Group 1 = RTTs using the tool; Group 2: RTTs using the tool limitedly or not) for each of the 10 digital tools. A paired samples *t*-test was used to identify differences between groups for the overall technostress score and the sub-scores (techno-overload, techno-insecurity, techno-uncertainty, techno-complexity).

Next, a linear regression model was performed three times (i.e. each time different predictor variables were used to investigate the contributions of the different predictors (Fig. 1)) for each of the key job performance indicators (Job Satisfaction, Job Engagement and Turnover Intention). First using the overall technostress score as the predictive value. Afterwards, the job autonomy, IWB score and the following combinations (technostress x IWB and Job Autonomy x IWB) were added as predictor variables in the linear regression model using a stepwise approach for each of the key job performance indicators. Finally, dummy variables for each of the control variables (age, working location, working hours, job role and working experience) were created and added as predictors in the stepwise linear regression model.

#### Future perspective

As part of the survey the RTTs were presented with a future view of their role (i.e. taking the expected technological advancements into account). The paired samples T-test was performed to identify pre-to-post (i.e. the score before and after the expected technological advancements) differences for the Job Engagement, Job Satisfaction and Turnover Intention. Subsequently, a linear regression model was performed to predict the pre-to-post difference for each of the key job performance indicators using the Job Autonomy and IWB score as predictors. Finally, dummy variables for each of the control variables (age, working location, working hours, job role and working experience) were created and added as predictors in the stepwise linear regression model. Statistical analyses were performed using SPSS (version 28, SPSS Inc., Irvine, CA) and the significance level was set at p = 0.05.

## Results

### Participant characteristics

A total of 265 RTTs completed the online questionnaire reaching all radiotherapy centers in the Netherlands (estimated response rate of 33 %). Most of the respondents were female (84.5 %) and respondents covered all different age groups (Table 1). Moreover, the RTTs typically worked 33–40 h (41.5 %) a week as an all-round RTT (45.7 %) and differed in working experience (Table 1).

**Table 1:** Demographic Characteristics of the 265 respondents of the present study.

**Variable**	**Count** (Total = 265)	**Percentage**
**Gender**		
Female	224	84.5
Male	39	14.7
Other	2	0.8

**Age**		
18-25 Years	32	12.1
26-35 Years	98	37
36-45 Years	78	29.4
46-55 Years	30	11.3
> 55 Years	27	10.2

**Working hours**		
0-8 Hours	2	0.8
9-16 Hours	3	1.1
17-24 Hours	55	20.8
25-32 Hours	95	35.8
33-40 Hours	110	41.5

**Working Experience**		
0-5 Years	77	29.1
6-10 Years	56	21.1
11-15 Years	31	11.7
16-20 Years	32	12.1
>20 Years	69	26

**Job Role**		
Planning RTT	31	11.7
All-round RTT	121	45.7
Device RTT	80	30.2
Other	33	12.5

### Current use of digital tools versus job performance indicators

On average the RTTs used 3.5 (± 2.2) out of the 10 tools in their daily work routine (Table 2). The four most commonly used tools were: 1) The use of an electronic medical record; 2) Software to automate the preparation phase (e.g. scripting); 3) Software to automatically position the patient; 4) Automatic contouring software. No significant linear regression model could be obtained for the Job Satisfaction (p = 0.278), Job Engagement (p = 0.141) and Turnover Intention (p = 0.059) using the count of digital tools as predictive variable.

**Table 2:** Overall scores (±SD) for the different outcome parameters. For the technostress parameters were scored on a five-point Likert scale (1 = strongly disagree; 2 = disagree; 3 = neutral; 4 = agree; 5 = strongly agree).

**Variable**	**Score** (n = 265)
**Count of digital tools** (max. score = 10)	3.5 ± 2.2
**Overall technostress** (max. score = 5)	2.41 ± 0.47
Techno-overload	2.73 ± 0.62
Techno-insecurity	1.92 ± 0.72
Techno-uncertainty	3.98 ± 0.65
Techno-complexity	2.07 ± 0.71
**Job Autonomy** (max. score = 5)	3.3 ± 0.4
**Job Satisfaction** (max. score = 5)	4.1 ± 0.6
**Job Engagement** (max. score = 5)	3.6 ± 0.4
**Turnover Intention** (max. score = 5)	2.0 ± 1.0

### Technostress and Job Autonomy

#### Overall scores

The RTTs scored neutral in terms of the presence of technostress, as indicated by an overall technostress score of 2.41, whereas the scores for the different sub-categories varied (Table 2). The scores for the Turnover Intention, Job Autonomy, Job Engagement and Job Satisfaction ranged from 2.0 to 4.1 (Table 2).

#### Technostress and Job Autonomy versus digital tools

No significant linear regression model could be obtained for the overall technostress (p = 0.946) using the count of digital tools as predictive variable. Furthermore, there was no significant (p = 0.089 – 0.991) difference in the level of technostress when we compared the 10 tools separately between RTTs using the tool versus the RTTs who did not use the tool. Only when comparing the different sub-categories of technostress (i.e. techno-overload, techno-insecurity, techno-uncertainty, techno-complexity) significant differences between RTTs who used the specific tool regularly and RTTs who did not or only limitedly use the specific tool were identified (see Supplementary Table 2).

A significant linear regression model (p = 0.006) was obtained for the Job Autonomy score using the count of digital tools as predictive variable. Approximately 2 % of the variance could be predicted by the count of digital tools (Adjusted R-square = 0.024).

#### Technostress and Job autonomy versus key job performance indicators

A significant relationship between the overall technostress and the key job performance indicators was identified (Table 3). The significant linear regression models revealed that the technostress could predict approximately 6 % of the variance for the Job Satisfaction, 2 % of the variance for the Job Engagement and approximately 6 % of the variance of the Turnover Intention (Table 3). Next the Job Autonomy, IWB score and the following combinations (technostress x IWB and Job Autonomy x IWB) were added as predictor variables in the model and the stepwise procedure revealed that for each of the key job performance indicators a significant model could be obtained with a higher predictive value (Table 3). Finally, when adding control variables to the linear regression models the predictive value once again increased (adjusted R-square: Job Satisfaction: 0.22; Job Engagement: 0.15; Turnover Intention: 0.26) (Table 3).

**Table 3:** Overview of the linear regression models with the key job performance indicators as dependent variables and the listed variables as independent variables.

**Variable**	**Job Satisfaction**			**Job Engagement**			**Turnover Intention**		
	RC	*p value*	Adj. R^2^	RC	*p value*	adj R^2^	RC	*p value*	adj R^2^
**Model 1**		**<0.001**	**0.062**		**0.013**	**0.020**		**<0.001**	**0.056**
Technostress score	−0.33	<0.001		−0.13	0.013		0.53	<0.001	
Constant	4.87	<0.001		3.94	0<.001		0.73	0.022	

**Model 2**		**<0.001**	**0.199**		**<0.001**	**0.108**		**<0.001**	**0.154**
Technostress score	−0.23	0.002		−	−		−1.26	0.007	
Job Autonomy	0.58	<0.001		0.34	<0.001		−0.66	<0.001	
IWB score	−	−		−	−		−1.19	0.001	
Technostress x IWB score	−	−		−	−		0.58	<0.001	
Job Autonomy x IWB score	−	−		−	−		−	−	
Constant	2.69	<0.001		2.49	<0.001		6.64	<0.001	

**Model 3**		**<0.001**	**0.224**		**<0.001**	**0.145**		**<0.001**	**0.257**
Technostress score	−0.22	<0.001		−	−		−1.23	0.006	
Job Autonomy	0.66	0.003		0.31	<0.001		−0.76	<0.001	
IWB score	−	−		−	−		−1.25	<0.001	
Technostress x IWB score	−	−		−	−		0.59	<0.001	
Working experience 16–20 years	−	−		−	−		−0.44	0.013	
Working experience > 20 years	0.18	0.019		−	−		−0.50	<0.001	
Job role: Planning RTT	−0.24	0.026		−	−		−	−	
Age: 18–25 years	−	−		0.20	0.007		−	−	
Working location: Utrecht	−	−		−0.34	0.021		1.25	<0.001	
Working location: Ams. UMC	−	−		−	−		0.86	0.006	
Working hours: 17–24	−	−		−	−		0.34	0.017	
Constant	2.39	<0.001		2.57	<0.001		7.07	<0.001	

### Future perspective

The paired samples T-test identified a significant difference for the Job Satisfaction (p < 0.001) and Turnover Intention (p < 0.001) between the current score and future expectation of the RTTs (Fig. 2). No significant difference for the Job Engagement was identified. The Job Autonomy and IWB scores did not significantly (p = 0.181–––0.254) predict the difference in the current score and future expectation of the RTT’s Job Satisfaction and Turnover Intention scores.

## Discussion

In the present study we distributed a survey amongst the RTTs in the Netherlands and sought to establish 1) the impact of the currently used digital tools on the RTT’s key job performance indicators (Job Engagement, Job Satisfaction and Turnover Intentions) and 2) the impact of the expected digital transformation in radiotherapy on the RTT’s key job performance indicators.

### Use of digital tools

Extensive use of digital tools did not negatively affect the key job performance indicators of the RTTs. Additionally, RTTs using a higher number of digital tools did not display greater levels of technostress. This contradicts with a recent article showing that digital overload has significant negative effects on Job Satisfaction [30]. This article of Fleisher et al. [30] highlights that serving multiple ICT tools simultaneously is likely to raise disturbance frequency and to force employees to interrupt their workflow [31,32]. The fact that Dutch RTTs are not influenced by the use of a high number of digital tools can be explained by the fact that radiotherapy is a specialty that has historically evolved by taking advantage of technology [13,33] and, as a result, RTTs in the Netherlands are used to working with different digital tools. In fact, RTTs that used a high number of tools even showed a reduced techno-complexity score, which indicates that they are less prone to experience technostress.

### Technostress and Job autonomy versus key job performance indicators

Even though the experienced technostress had a significant negative impact on the key job performance indicators of the RTTs, less than 7 % of the variance in the key job performance indicators could be explained by the technostress scores, highlighting that this relationship is neglectable for the RTTs in the present study. Job Autonomy and the IWB played a far greater role in explaining the Job Satisfaction and Job Engagement scores, whereas the control variables (i.e. working location and experience) primarily determined whether someone would consider leaving their job. The importance of Job Autonomy has been emphasized in the literature in which the negative impact of digital overload on Job Satisfaction is partially conveyed through a loss of Job Autonomy [30]. Indeed, according to the self-determination theory autonomy is often regarded as the most essential need and settings that meet the individual demands for autonomy induce motivational regulation styles that are important for enjoyment and satisfaction [30,34]. This highlights that instead of focusing on techno-stressors, it is more important to make sure that a RTT feels that they have the appropriate skills to perform and fulfill the tasks assigned to them and have decision-making authority in fulfilling these tasks (i.e. perceives high levels of Job Autonomy) [22]. Therefore it is important to continue to invest in education and training programs to strengthen employees’ digital resilience [28], thus encouraging digital tool usage in a self-paced and self-determined manner while increasing decision authority that will result in increased Job Autonomy levels.

### Future perspective

When presented with the future perspective of their job role, the RTTs reported small declines in the key job performance indicators. However, the overall scores remained ‘positive’ as the RTTs were still satisfied and engaged in their job and did not show an intention to leave their job after the predicted digitalization. This highlights that RTTs are willing to accept that, for example, automation and digitalization could simplify or even make parts of their job redundant (e.g. continued automation of the treatment planning process) [10]. Again this can be explained by the fact that radiotherapy takes advantage of technology [13] and RTTs are used to new technological implementations. As mentioned before, education and training together with a focus on increasing decision authority of the RTT is essential to ensure RTTs are well-prepared to meet the demands of modern radiation therapy, as insufficient training could hinder the adoption of innovative techniques [33]. Overall this emphasizes that although implementing digital tools may refocus tasks performed by the RTTs, the potential benefit to counteract the staff shortages and high levels of burnout and staff turnover outweighs these changes in job role [9].

### Limitations

A limitation of this study is the limited generalizability due to the potential between country differences in terms of the RTT role (e.g. different responsibilities) and training received by the RTT. Although, this is an important factor to consider, it should be noted that within the Netherlands similar differences between centers are present. Furthermore, the conclusions drawn regarding the nature of the profession (i.e. RTTs are used to working with digital tools) and the importance of having decision-making authority are relevant for RTTs in all countries.

## Conclusion

In conclusion, the findings of the present study demonstrate that the current performance of the RTT is not affected by the number of digital tools used, most likely as RTTs are used to working with digital tools. Furthermore, the RTTs show no indications of technostress. After the predicted digital transformation, the RTT’s key job performance indicators declined, but remained ‘positive’ as the RTTs were still satisfied and engaged with their job and did not intend to leave their job. Other factors like age and working location play a greater role in predicting whether the RTT would leave their job. Therefore, instead of focusing on the number of digital tools or technostress, it is crucial to make sure that RTTs are confident that they have the appropriate training/skills to perform the tasks assigned to them and have decision-making authority in fulfilling these tasks (i.e. perceive high levels of Job Autonomy). This should be considered before the implementation of a new digital tool.

## CRediT authorship contribution statement

**Thijs Ackermans:** Formal analysis, Investigation, Writing – original draft, Methodology. **Paul Cremers:** Conceptualization, Supervision, Data curation, Writing – review & editing. **Janne Dingemans:** Methodology, Writing – original draft, Visualization, Investigation. **Carol Ou:** Methodology, Visualization, Writing – review & editing. **Marcel Verheij:** Methodology, Writing – review & editing. **Maria Jacobs:** Conceptualization, Supervision, Writing – review & editing.

## Declaration of competing interest

The authors declare that they have no known competing financial interests or personal relationships that could have appeared to influence the work reported in this paper.
