# Supplementary material for: The impact of digital tools and the expected digital transformation in radiotherapy on Dutch radiation therapists (RTTs)
Source: Tech Innov Patient Support Radiat Oncol. 2025 Jun 4;34:100319. doi: 10.1016/j.tipsro.2025.100319 (PMC12173727; doi:10.1016/j.tipsro.2025.100319)
Supplement: Supplementary Data 1 [file mmc1.docx]

**Supplementary Table 1.** Overview of the questionnaire used.

| **Persoonlijke gegevens** | |
| --- | --- |
| 1 | In welke leeftijdscategorie val je? *(18-25j; 26-35j; 36-45j; 46-55j; >55j)* |
| 2 | Wat is je geslacht? *(Man; Vrouw; Anders)* |
| 3 | Wat is je huidige rol als MBB'er werkzaam in de radiotherapie? *(All-round laborant; Plannings laborant; Toestel laborant; Anders namelijk …)* |
| 4 | Hoe lang ben je al werkzaam in deze rol als MBB'er in de radiotherapie?*(0-5j; 6-10j; 11-15j; 16-20j; or >20j)* |
| 5 | Hoeveel uren werk je per week in deze rol? *(0-8u; 9-16u; 17-24u; 25-32u; or 33-40u)* |
| 6 | In welk centrum ben je werkzaam als MBB'er in de radiotherapie?  *Open vraag* |
| **Taakkarakteristieken** (Helemaal-Oneens; Oneens; Neutraal; Eens; Helemaal Eens) | |
| 7 | Zelf te beslissen hoe ik mijn werk indeel. |
| 8 | Zelf te beslissen in welke volgorde dingen gedaan worden op het werk. |
| 9 | Om zelf te plannen hoe ik mijn werk doe. |
| 10 | Brengt veel afwisselende taken met zich mee. |
| 11 | Bestaat uit het doen van verschillende dingen. |
| 12 | Vereist het uitvoeren van een breed scala aan taken. |
| 13 | Biedt afwisseling in taken. |
| 14 | Omvat het afmaken van een herkenbaar stuk werk met een duidelijk begin en eind. |
| 15 | Is zo georganiseerd, dat ik een compleet stuk werk van begin tot eind kan afmaken. |
| 16 | Biedt mij de kans werk waar ik aan begin, volledig af te maken. |
| 17 | Mijn baan biedt mij de kans mijn eigen initiatief of oordeel te volgen in hoe ik mijn werk uitvoer. |
| 18 | In mijn baan kan ik veel beslissingen zelf nemen. |
| 19 | Mijn baan biedt in belangrijke mate zelfstandigheid om beslissingen te nemen. |
| 20 | In mijn baan kan ik zelf beslissen welke methoden ik gebruik om mijn werk af te maken. |
| 21 | Mijn baan biedt mij een behoorlijke mate van onafhankelijkheid en vrijheid in hoe ik mijn werk doe. |
| 22 | Mijn baan biedt mij de mogelijkheid te beslissen hoe ik te werk ga. |
| 23 | Het is waarschijnlijk dat de resultaten van mijn werk het leven van andere mensen in belangrijke mate beïnvloeden. |
| 24 | Mijn baan zelf is erg belangrijk in een groter geheel. |
| 25 | Mijn baan heeft een grote invloed op mensen buiten de organisatie. |
| 26 | De werkzaamheden die worden uitgevoerd voor mijn baan, hebben in belangrijke mate invloed op mensen buiten de organisatie. |
| 27 | Mijn werkzaamheden bieden direct duidelijke informatie over hoe effectief ik mijn werk doe, zowel in termen van kwaliteit als hoeveelheid. |
| 28 | Mijn werk zelf biedt feedback over mijn prestaties. |
| 29 | Het werk zelf biedt informatie over mijn prestaties. |
| **Werkprestatie indicatoren**(Helemaal-Oneens; Oneens; Neutraal; Eens; Helemaal Eens) | |
| 30 | Alles overziend ben ik tevreden met mijn werk. |
| 31 | Ik denk er sterk over deze organisatie binnen twaalf maanden te verlaten. |
| 32 | Ik overweeg te stoppen in de functie als laborant.  Ik barst van de energie tijdens mijn werk. |
| 33 |  |
| 34 | Ik voel me sterk en energiek tijdens mijn werk. |
| 35 | Ik ben enthousiast over mijn werk. |
| 36 | Mijn werk inspireert mij. |
| 37 | Ik heb 's morgens zin om naar mijn werk te gaan. |
| 38 | Ik ben blij als ik hard aan het werk ben. |
| 39 | Ik ben trots op het werk dat ik doe. |
| 40 | Ik ben ondergedompeld in mijn werk. |
| 41 | Ik verlies mezelf in mijn werk. |
| **Digitale Tools** (Nooit; Zelden; Soms; Vaak; Altijd) | |
| *In welke mate gebruik je de volgende digitale tools om je taken uit te voeren als MBB'er werkzaam in de radiotherapie:* | |
| 42 | Software om routinematig het voorbereidingsproces te automatiseren (Zoals, Scripting). |
| 43 | Software om CTs automatisch in te lezen. |
| 44 | Software om contouren te maken (Auto-contouring of Automatisch intekenen). |
| 45 | Software om het behandelplan te maken (Automatic planning). |
| 46 | Software om de kwaliteit van mijn behandelplan te vergelijken met de database (Plan optimalisatie of Quality check). |
| 47 | Software om een afwijking van de behandelprocedure te detecteren (Automatic detection of ‘deviations’). |
| 48 | Software om de patiënt te positioneren op de behandeltafel. |
| 49 | Software of applicaties om het elektronisch gezondheidsdossier (EHR) van de patiënt bij te werken (zodat dit niet op papier gebeurd). |
| 50 | Software om na de behandeling een rapport op te stellen (Data koppeling). |
| 51 | Software om een 3D reconstructie te maken. |
| **Digitale Tools** (Helemaal-Oneens; Oneens; Neutraal; Eens; Helemaal Eens) | |
| *Ik word door digitale tools gedwongen om:* | |
| 52 | Sneller te werken. |
| 53 | Meer werk te doen dan ik aankan. |
| 54 | Te werken met strakke tijdsplanningen. |
| 55 | Mijn werkgewoontes te veranderen door nieuwe technologieën toe te passen. |
| 56 | Om te gaan met een hogere werkdruk vanwege de toegenomen complexiteit van technologieën. |
| 57 | Dat mijn baan voortdurend op het spel staat. |
| 58 | De behoefte om voortdurend mijn vaardigheden te ontwikkelen om te voorkomen dat ik word vervangen. |
| 59 | Me voortdurend bedreigd door nieuwe collega's met betere ICT-vaardigheden. |
| 60 | In ons bedrijf zijn er altijd nieuwe ontwikkelingen in de digitale tools die we gebruiken. |
| 61 | Ik begrijp te weinig van de nieuwste digitale tools/innovaties om mijn werk naar tevredenheid uit te voeren. |
| 62 | Ik heb niet voldoende tijd om mijn technologische-vaardigheden te ontwikkelen. |
| 63 | Ik heb veel tijd nodig om het gebruik van nieuwe digitale tools/innovaties te begrijpen. |
| 64 | Ik vind het vaak te complex om nieuwe digitale tools/innovaties te begrijpen en te gebruiken. |
| **Innovaties** (Helemaal-Oneens; Oneens; Neutraal; Eens; Helemaal Eens) | |
| 65 | Ik vind dat nieuwe werknemers bij dit bedrijf meer over digitale tools/innovaties weten dan ik. |
| 66 | Ik bedenk nieuwe ideeën voor moeilijke problemen. |
| 67 | Ik ga op zoek naar nieuwe manieren of instrumenten voor mijn werk om tot vernieuwing te komen. |
| 68 | Ik ontwikkel originele oplossingen voor problemen. |
| 69 | Ik creëer draagvlak voor innovatieve ideeën. |
| 70 | Ik probeer steun te krijgen wanneer ik iets nieuws bedenk. |
| 71 | Ik maak leidinggevenden van de organisatie enthousiast voor innovatieve ideeën. |
| 72 | Ik vertaal innovatieve ideeën naar bruikbare toepassingen. |
| 73 | Ik werk systematisch aan het introduceren van innovatieve ideeën. |
| 74 | Ik ga na in hoeverre innovatieve ideeën waardevol zijn geweest. |
| **Toekomst Perspectief** (Helemaal-Oneens; Oneens; Neutraal; Eens; Helemaal Eens) | |
| *Als gevolg van de aankomende digitale transformatie is het waarschijnlijk dat:* | |
| 75 | Ik alles overziend tevreden ben met mijn werk. |
| 76 | Ik er sterk over denk om deze organisatie binnen twaalf maanden te verlaten. |
| 77 | Ik overweeg te stoppen in de functie als laborant. |
| 78 | Ik mezelf verlies in mijn werk. |
| 79 | Ik barst van de energie tijdens mijn werk. |
| 80 | Ik me sterk en energiek voel tijdens mijn werk. |
| 81 | Ik enthousiast ben over mijn werk. |
| 82 | Mijn werk mij inspireert. |
| 83 | Ik 's morgens zin heb om naar mijn werk te gaan. |
| 84 | Ik blij ben als ik hard aan het werk ben. |
| 85 | Ik trots ben op het werk dat ik doe. |
| 86 | Ik ondergedompeld ben in mijn werk. |

**Supplementary Table 2.** Overview of the statistical analysis of the comparison of the different sub-categories of technostress (i.e. techno-overload, techno-insecurity, techno-uncertainty, techno-complexity) between RTTs who used the specific tool and RTTs who limited or did not use the specific tool were identified.

|  | **Overall technostress** (max. 5) | **Techno-overload** (max. 5) | **Techno-insecurity** (max. 5) | **Techno-uncertainty** (max. 5) | **Techno-complexity** (max. 5) |
| --- | --- | --- | --- | --- | --- |
| 1. Software to automate the preparation phase (e.g. scripting).  ***U:*** *n=117;* ***LN-U:*** *n= 148* | **U:** 2.43 ± 0.49  **LN-U:** 2.39 ± 0.56  *p = 0.539* | **U:** 2.78 ± 0.62  **LN-U:** 2.68 ± 0.62  *p* *= 0.221* | **U:** 1.94 ± 0.81  **LN-U:** 1.90 ± 0.64  *p* = 0.590 | **U:** 4.0 ± 0.59  **LN-U:** 3.9 ± 0.69  *p = 0.169* | **U:** 2.04 ± 0.72  **LN-U:** 2.09 ± 0.70  *p = 0.619* |
| 2. Software to automatically import CTs.  ***U:*** *n=72;* ***LN-U:*** *n= 193* | **U:** 2.39 ± 0.44  **LN-U:** 2.41 ± 0.49  *p* = *0.707* | **U:** 2.76 ± 0.61  **LN-U:** 2.71 ± 0.62  *p* *= 0.597* | **U:** 1.83 ± 0.69  **LN-U:** 1.96 ± 0.73  *p* *= 0.196* | **U:** 4.06 ± 0.60  **LN-U:** 3.95 ± 0.66  *p* *= 0.254* | **U:** 2.03 ± 0.60  **LN-U:** 2.08 ± 0.75  *p = 0.555* |
| 3. Software to optimize the treatment plan  ***U:*** *n=56;* ***LN-U:*** *n= 209* | **U:** 2.37 ± 0.44  **LN-U:** 2.42 ± 0.48  *p* = *0.459* | **U:** 2.76 ± 0.62  **LN-U:** 2.72 ± 0.62  *p* = *0.666* | **U:** 1.89 ± 0.63  **LN-U:** 1.93 ± 0.74  *p = 0.680* | **U:** 4.04 ± 0.57  **LN-U:** 3.97 ± 0.67  *p = 0.479* | **U:** 1.93 ± 0.60  **LN-U:** 2.10 ± 0.73  *p = 0.100* |
| 4. Software to automatically position the patient.  ***U:*** *n=108;* ***LN-U:*** *n= 157* | **U:** 2.41 ± 0.46  **LN-U:** 2.41 ± 0.49  *p* = *0.991* | **U:** 2.76 ± 0.62  **LN-U:** 2.70 ± 0.62  *p* = *0.390* | **U:** 1.86 ± 0.71  **LN-U:** 1.96 ± 0.72  *p = 0.277* | **U:** 4.05 ± 0.63  **LN-U:** 3.94 ± 0.66  *p = 0.175* | **U:** 2.05 ± 0.80  **LN-U:** 2.08 ± 0.64  *p* = *0.718* |
| 5. Automatic contouring software.  ***U:*** *n=107;* ***LN-U:*** *n= 158* | **U:** 2.42 ± 0.46  **LN-U:** 2.40 ± 0.49  *p* = *0.623* | **U:** 2.80 ± 0.67  **LN-U:** 2.68 ± 0.58  *p* *= 0.116* | **U:** 1.92 ± 0.66  **LN-U:** 1.92 ± 0.75  *p = 0.955* | **U:** 4.07 ± 0.61  **LN-U:** 3.92 ± 0.67  *p = 0.053* | **U:** 2.03 ± 0.75  **LN-U:** 2.09 ± 0.65  *p* = *0.440* |
| 6. Software to automate the development of the treatment plan.  ***U:*** *n=208;* ***LN-U:*** *n= 57* | **U:** 2.48 ± 0.48  **LN-U:** 2.39 ± 0.47  *p* = *0.188* | **U:** 2.92 ± 0.58  **LN-U:** 2.67 ± 0.62  *p = 0.009* | **U:** 1.98 ± 0.76  **LN-U:** 1.91 ± 0.70  *p = 0.517* | **U:** 4.05 ± 0.61  **LN-U:** 3.96 ± 0.66  *p = 0.348* | **U:** 2.04 ± 0.68  **LN-U:** 2.08 ± 0.72  *p* = *0.701* |
| 7. Software to automatically detect deviations.  ***U:*** *n=40;* ***LN-U:*** *n= 225* | **U:** 2.42 ± 0.50  **LN-U:** 2.41 ± 0.47  *p* = *0.844* | **U:** 2.77 ± 0.60  **LN-U:** 2.72 ± 0.62  *p* *= 0.661* | **U:** 1.88 ± 0.71  **LN-U:** 1.93 ± 0.72  *p = 0.712* | **U:** 4.20 ± 0.69  **LN-U:** 3.94 ± 0.63  *p = 0.020* | **U:** 2.05 ± 0.76  **LN-U:** 2.07 ± 0.70  *p* = *0.831* |
| 8. Software for automatic reporting (data coupling).  ***U:*** *n=172;* ***LN-U:*** *n= 193* | **U:** 2.39 ± 0.46  **LN-U:** 2.42 ± 0.48  *p* = *0.602* | **U:** 2.87 ± 0.67  **LN-U:** 2.65 ± 0.58  *p* *= 0.005* | **U:** 1.87 ± 0.76  **LN-U:** 1.95 ± 0.69  *p = 0.362* | **U:** 4.01 ± 0.70  **LN-U:** 3.97 ± 0.62  *p* = *0.585* | **U:** 1.89 ± 0.61  **LN-U:** 2.16 ± 0.74  *p* = *0.003* |
| 9. Software to automatically make 3D reconstruction.  ***U:*** *n=102;* ***LN-U:*** *n= 163* | **U:** 2.44 ± 0.46  **LN-U:** 2.38 ± 0.48  *p* = *0.318* | **U:** 2.83 ± 0.68  **LN-U:** 2.66 ± 0.57  *p = 0.027* | **U:** 1.92 ± 0.75  **LN-U:** 1.93 ± 0.70  *p = 0.900* | **U:** 4.05 ± 0.64  **LN-U:** 3.94 ± 0.65  *p* = *0.178* | **U:** 2.05 ± 0.68  **LN-U:** 2.07 ± 0.73  *p* = *0.824* |
| 10. The use of an electronic medical record.  ***U:*** *n=188;* ***LN-U:*** *n= 77* | **U:** 2.38 ± 0.48  **LN-U:** 2.49 ± 0.46  *p* = *0.089* | **U:** 2.74 ± 0.63  **LN-U:** 2.70 ± 0.61  *p* *= 0.656* | **U:** 1.87 ± 0.70  **LN-U:** 2.06 ± 0.75  *p = 0.051* | **U:** 4.00 ± 0.69  **LN-U:** 3.94 ± 0.52  *p* = *0.460* | **U:** 2.00 ± 0.69  **LN-U:** 2.24 ± 0.73  *p* = *0.011* |

***U*** *= people Using the tool****; NL-U****= people Using the tool Limited or Not*
